# PI3K-dependent GAB1/Erk phosphorylation renders head and neck squamous cell carcinoma sensitive to PI3Kα inhibitors

**DOI:** 10.1038/s41419-025-07767-x

**Published:** 2025-06-18

**Authors:** Xu Zhang, Jiao Xu, Xuan Wang, Lan Xu, Xi Zhang, Yi Wang, Shujuan Jiang, Yixiang Zhang, Jian Ding, Chen Qing, Linghua Meng

**Affiliations:** 1 Division of Anti-tumor Pharmacology, State Key Laboratory of Chemical Biology, Shanghai Institute of Materia Medica, Chinese Academy of Sciences, Shanghai, China; 2 School of Pharmaceutical Sciences & Yunnan Key Laboratory of Pharmacology for Natural Products, Kunming Medical University, Yunnan, China; 3 School of Life Science and Technology, ShanghaiTech University, Shanghai, China; 4 Haihe Biopharma Co. Ltd, Shanghai, China; 5 Division of Anti-tumor Pharmacology, State Key Laboratory of Drug Research, Shanghai Institute of Materia Medica, Chinese Academy of Sciences, Shanghai, China; 6 University of Chinese Academy of Sciences, Beijing, China

**Keywords:** Targeted therapies, Head and neck cancer, Clinical pharmacology

## Abstract

The hyperactivation of the PI3K pathway in head and neck squamous cell carcinoma (HNSCC) suggests that targeting PI3K is a potential therapeutic strategy. CYH33 is a novel PI3Kα-selective inhibitor discovered by our group, which is currently undergoing a phase I clinical trial (NCT03544905) for the treatment of advanced solid tumors including HNSCC. However, there is an urgent need to elucidate its mechanism of action and improve its efficacy against HNSCC. In this study, we found that CYH33 displayed promising but variable therapeutic activity against HNSCC. Inhibition of PI3K/Akt pathway by CYH33 was not sufficient for its activity against HNSCC. Tandem-Mass-Tag (TMT) phosphoproteomics were performed to reveal comprehensive regulation of kinome by CYH33. Particularly, attenuation of Erk phosphorylation was associated with the sensitivity of HNSCC cells to CYH33. Mechanistically, inhibition of PI3K by CYH33 blocked the PIP3 production and attenuated the membrane localization and phosphorylation of GAB1, resulting in reduced Erk phosphorylation and ultimately inhibition of cell proliferation in sensitive HNSCC cells. Meanwhile, activation of EGFR induced GAB1 phosphorylation independent of PI3K in HNSCC cells. Concurrent inhibition of EGFR synergistically potentiated the activity of CYH33 against HNSCC. These findings revealed the insight mechanism of CYH33 against HNSCC and provided rational combination regimen for HNSCC treatment.

## Introduction

Head and neck cancer is the seventh most common cancer in the world, of which about 90% are head and neck squamous cell carcinoma (HNSCC) [1]. HNSCC originates from the squamous epithelium lining the mucosal surfaces of the head and neck region, including the oral cavity, pharynx, and larynx [2]. Currently, the therapeutic options for HNSCC remain limited. Drugs approved by the Food and Drug Administration (FDA) for HNSCC treatment include chemotherapeutic agents (cisplatin, 5-fluorouracil, methotrexate, and docetaxel), epidermal growth factor receptor (EGFR) inhibitor Cetuximab and immune checkpoint inhibitors (Nivolumab and Pembrolizumab) [3]. However, a considerable proportion of patients will encounter acquired resistance after chemotherapy and only a subset of patients will respond to either Cetuximab or check point inhibitors [4–6]. The overall 5-year survival rate of HNSCC patients is less than 50% up to date [7]. HNSCC remains a critical unmet medical need on a global scale.

Phosphatidylinositol 3-kinase (PI3K) serves as a key integrator of signals from diverse environmental factors, playing a crucial role in regulating cellular processes such as metabolism, growth, apoptosis, and cytoskeletal organization [8, 9]. Large scale next-generation sequencing has revealed that PI3K is the most commonly altered pathway in HNSCC [10, 11]. Alterations of *PIK3CA* encoding the catalytic subunit p110α have been found in 48% of HNSCC patients [12]. Moreover, hyper-activation of receptor tyrosine kinases (RTKs) also results in active PI3K signaling [13]. Selective targeting PI3Kα has emerged as a potential approach for the therapy of HNSCC. Alpelisib, the first PI3Kα selective inhibitor approved by the FDA, is currently tested in HNSCC patients either as a monotherapy or in combination with Cetuximab. The pan-PI3K inhibitor buparlisib was evaluated in combination with paclitaxel in a phase II trial for the treatment of recurrent or metastatic HNSCC patients, demonstrating manageable safety profile and preliminary anti-HNSCC activity [14]. However, over 50% of HNSCC patients still failed to achieve substantial clinical remission, highlighting the necessity to develop new PI3K inhibitors with improved efficacy. On the other hand, comprehensive elucidation of the molecular mechanisms underlying the anti-HNSCC activity of PI3K inhibitors remain largely undefined, which is fundamental to uncover novel therapeutic strategies to optimize the clinical efficacy.

CYH33 is a novel PI3Kα-selective inhibitor discovered in our previous work, which is in phase I/II clinical trials for the therapy of advanced solid tumors including HNSCC (NCT05043922, NCT04586335 and NCT03544905) [15, 16]. Data obtained from the completed phase Ia trial indicated that CYH33 demonstrated a manageable safety profile, linear pharmacokinetics, and encouraging preliminary anti-tumor activity [17]. Complete or partial response was observed in breast cancer patients harboring *PIK3CA* mutation [18]. In this study, we found CYH33 displayed promising but variable activity against HNSCC cells. Inhibition of PI3K/Akt pathway by CYH33 was not sufficient for its activity against HNSCC. Tandem-Mass-Tag (TMT) proteomics and phosphoproteomics revealed the comprehensive regulation of kinome by CYH33. We discovered that attenuation of PI3K-dependent GAB1/Erk phosphorylation was associated with the anti-HNSCC activity of CYH33. Simultaneous inhibition of GAB1 phosphorylation independent of PI3K potentiated its activity against HNSCC. These findings revealed the insight mechanism of CYH33 against HNSCC and provided rational combination regimen for HNSCC.

## Materials and methods

### Cell lines and cell culture

The HNSCC cells CNE-1, SUNE-1, C666 were kindly provided by Zhongshan Hospital, Fudan University. The HNSCC cells CNE-2Z, RPMI-2650 were purchased from Cobioer Biotechnology Co., Ltd. The HNSCC cells HN6, HN30, HN12, HN4 were kindly provided by Shanghai Chest Hospital, Shanghai Jiao Tong University. The HNSCC cells SCC-9, SCC-25, SCC-154, SCC-90, SCC-4, SCC-152, 2A3, FaDu, KB were purchased from ATCC. The HNSCC cells Tca-8113 were kindly provided by Shanghai Ninth People’s Hospital, Shanghai Jiao Tong University. Cell lines employed were authenticated by analyzing short tandem repeats at Genesky Biotechnologies Inc. (Shanghai, China). Cells were cultured with recommended medium in a humidified atmosphere containing 5% CO2 at 37 °C. Cisplatin-resistant HN4 and HN30 cells were established by exposing cells to increasing concentrations of cisplatin as described previously [19].

### Regents

CYH33 was obtained from Shanghai HaiHe Biopharma Co., Ltd (Shanghai, China). Alpelisib, TGX-221, Linsitinib, Afatinib, Sapitinib, Capmatinib and Fexagratinib were purchased from Selleck Chemicals (Houston, USA). Cisplatin, Paclitaxel and U0126 were purchased from Meilunbio (Dalian, China). Cetuximab was purchased from Merck KGaA (Darmstadt, Germany). For experiments in vitro, all compounds were dissolved in dimethyl sulfoxide (DMSO, Sigma, St. Louis, USA) at 10 mM and stored at −20 °C. For animal studies, Cetuximab was dissolved in normal saline, while CYH33 were dissolved in normal saline containing 0.5% of Tween 80 (v/v; Sangon Biotech, Shanghai, China) and 1% of CMC-Na (m/v; Sinopharm, Beijing, China). Human IGF, FGF, EGF, NRG1 and HGF were purchased from Prepro Tech (New Jersey Rocky Hill, USA). All growth factors were dissolved in 0.1% BSA as stock solution and the aliquots were stored at −20 °C.

### Cell proliferation assay

Sulforhodamine B (SRB) assay was employed to evaluate cell proliferation as described previously [20]. The cell viability of RPMI-2650, HN12 and SCC-152 was measured with CellTiter-Glo assay (Promega Corporation, Madison, USA). The inhibitory rate was calculated using the formula: (OD_control cells_ − OD_treated cells_)/OD_control cells_ × 100%. IC_50_ value was computed by four parameter concentration response curves fitting with SoftMaxPro (Molecular Devices, USA).

### Western blotting

Cells or tumor tissues were lysed with radioimmunoprecipitation assay buffer (Beyotime, Shanghai, China, #P0013B) supplemented with protease inhibitors and phosphatase inhibitors (Beyotime, Shanghai, China, #P1006). Standard Western blotting was performed with antibodies against Akt (#4691), phospho-Akt (Ser473; #4060), S6K (#5707), phospho-S6K (Thr389; #9205), Erk (#4695), phospho-Erk (Thr185/Tyr187; #4370), GAB1 (#93804), phospho-GAB1 (Tyr 659; #12745), PI3Kα (#4249), PI3Kβ (#3011), PTEN (#9559) (Cell Signaling Technology, Danvers, USA) and GAPDH (#G8795) (Sigma, St. Louis, USA). Images were captured with the ChemiDoc Touch Imaging System (Bio-Rad, California, USA).

### Tandem-Mass-Tag proteomics/phosphoproteomics analysis

Protein extraction, digestion, TMT labeling, high pH reversed-phase liquid chromatography peptides fractionation and phosphopeptide enrichment were performed as described previously [21]. Raw files were processed by search against the UniProt/SwissProt Huamn database containing 75074 sequence entries using Maxquant (1.6.17.0) [22], with default settings for 6-plex TMT quantification. Trypsin/P was selected as the digestive enzyme with allowance of one missed cleavage. Minimum 7 amino acids for peptide, >2 peptides were required per protein. For peptide, protein and phosphosite identification, false discovery rate was set to 1%. TMT reporter ion intensity was used for quantification. Phosphoproteomics and proteomics data were analyzed in the R. The “clusterProfile” package in R was used to perform the Gene Ontology (GO) enrichment analysis. Kinase-Substrate Enrichment Analysis (KSEA) was performed with Omicsolution database (https://www.omicsolution.com/wkomics). The kinome tree was generated using Coral (http://phanstiel-lab.med.unc.edu/CORAL).

### Plasmid construction and transient transfection

Wild type GAB1 and its variants were constructed into pEGFP-N1 vector by Synbio Technologies (Suzhou, China). SUNE-1 cells seeded in 6-well plates were transfected with pEGFP-N1 expressing GAB1 or its variants using Lipofectamine 2000 (Invitrogen, Carisbad, CA, USA) following the manufacturer’s instructions. Human GAB1 or myristoylated GAB1 (Myr-GAB1) was cloned and ligated into the expressing vector pCDH-CMV-MCS-EF1-Puro purchased from Synbio Technologies (Suzhou, China).

### Virus production and cell infection

HEK293T cells were transfected with plasmids of interest, psPAX (Addgene, #12260) and pMD2.G (Addgene, #12259) using Lipofectamine 3000 (Invitrogen, Carlsbad, CA, USA) as the protocol provided by the manufacturer. Medium supernatant containing lentivirus was collected with a 0.45 μm filter 48 h after transfection. SUNE-1 cells were infected with the virus with the help of polybrene at 6 μg/mL (Sigma, St. Louis, USA). Cells were selected in the presence of puromycin (3 μg/mL).

### EGFP-GAB1 localization assay

SUNE-1 cells expressing different variants of EGFP-GAB1 were seeded on slides in 24-well plate. After indicated treatment, cells were fixed with 4% of paraformaldehyde and stained with DAPI. The slides were photographed with an Olympus BX51 fluorescent microscope (Olympus, Tokyo, Japan).

### Immunohistochemical staining

Immunohistochemical (IHC) staining was performed by Shanghai ZuoCheng Biotech (Shanghai, China). Antibodies against cleaved caspase-3 (#9661) or Ki-67 (12202) were purchased from Cell Signaling Technology. Then histological sections were scanned by NanoZoomer S210 (Hamamatsu, Japan).

### Animal studies

The protocol and procedures were approved and conducted in accordance with the Institutional Animal Care and Use Committee. Female BALB/c athymic nude mice aged 4–5 weeks were obtained from the Shanghai Institute of Materia Medica (Shanghai, China). All mice were housed in a specific pathogen-free facility at Shanghai Institute of Materia Medica Animal Resource Center. Mice were maintained under a 12-h light–dark cycle with free access to food and water. Xenografts derived from HN4 or SUNE-1 cells were established by subcutaneously injecting cells suspended in Matrigel into the right side of axillary. Tumor sections were cut into pieces of about 40 mm^3^ and then transplanted subcutaneously into mice. Xenografts derived from SUNE-1-V/SUNE-1-GAB1/SUNE-1-Myr-GAB1 cells were established by subcutaneously injecting cells suspended in Matrigel into the right side of axillary. When tumor volume reached about 150 mm^3^, mice were randomized and administered orally with vehicle control or indicated compounds once a day. Body weight was recorded with an electronic balance and tumor volume was measured using a microcaliper twice per week. The tumor volume (V) was calculated using the formula V = a^2^b/2, a and b represented the tumor’s width and length respectively. RTV was calculated by the formula RTV = V_t_/V_0_, where V_0_ represented the tumor volume at the beginning of treatment, and V_t_ represented the tumor volume upon treatment. Treatment to control (T/C) values were calculated using the formula T/C = RTV_treatment_/RTV_control_ × 100%.

### Combination analysis

The combinatorial effect in vitro was analyzed as described previously to determine the combination index (CI) [20]. CI < 0.80 indicates synergistic effect, CI = 0.80–1.20 indicates additive effect and CI > 1.20 indicates antagonistic effect. The combinatorial effect in vivo was evaluated by combination ratio with the Bliss independence model [23, 24]. Synergy, additive effect or antagonism was defined when the combination ratio was more than 1, 1, or lower than 1 respectively.

### Statistics

Experiments were repeated at least three times or otherwise stated, and data were presented as mean ± standard deviation (SD) or mean ± standard error of mean (SEM). Data were tested for normality using the Shapiro-Wilk test. Statistical analyses were performed using Prism 8 (GraphPad, La Jolla, USA). Statistical comparison was carried out with Student’s *t* test for two groups or one-way ANOVA followed by Tukey multiple group comparison tests for more than two groups. *p < 0.05, **p < 0.01, ****p < 0.0001.

## Results

### CYH33 displayed promising therapeutic activity against HNSCC

**Fig. 1:**
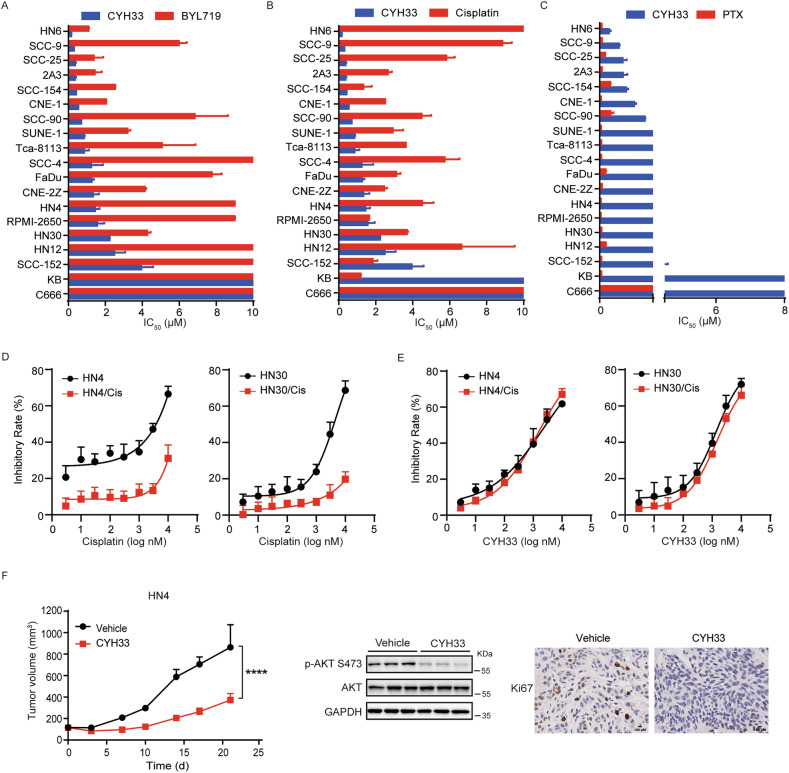
CYH33 displayed promising therapeutic activity against HNSCC. The half maximal inhibitory concentration (IC_50_) values of CYH33 and alpelisib (**A**)/cisplatin (**B**)/PTX (**C**) against the proliferation of 19 lines of HNSCC cells. Data were presented as mean + SD (*n* = 2). Parental and resistant cells were treated with cisplatin (**D**) or CYH33 (**E**) for 72 h and cell proliferation was measured by SRB assay (*n* = 3). **F** Randomly grouped BALB/c nude mice bearing HN4 xenografts were administrated orally with vehicle control or CYH33 (20 mg/kg) once a day for 21 days (*n* = 6). Tumor volume was measured twice a week. Data were presented as mean + SEM. Difference between groups was analyzed using two-tailed unpaired Student’s *t* test. ****p < 0.0001. Three representative tumors from each group were homogenized and then subjected to Western blot with the indicated antibodies. Tumor tissues were collected at the end of treatment to detect Ki-67. Scale bars, 100 μm. Representative images from each group are shown.

The hyperactivation of the PI3K pathway in HNSCC suggests that PI3K inhibition is a potential therapeutic strategy. To evaluate the therapeutic potential of the PI3Kα inhibitor CYH33 in HNSCC, we screened the anti-proliferative activity of CYH33 and alpelisib, the marketed PI3Kα-selective inhibitor, in 19 lines of well characterized HNSCC cells. As demonstrated in Fig. 1A, CYH33 significantly attenuated the proliferation of HNSCC cells with IC_50_ values below 2 μM in most of the tested cell lines. Alpelisib displayed a similar pattern against these cell lines, with IC_50_ values higher than those of CYH33. Cisplatin and paclitaxel have widely used for the treatment of HNSCC, which demonstrated potent anti-proliferative activity against HNSCC cells. (Fig. 1B, C). HNSCC patients are often refractory to cisplatin after initial treatment. HN4 and HN30 cells sensitive to cisplatin were exposed to gradient concentrations of cisplatin and cisplatin-resistant cell lines were established. As shown in Fig. 1D, cisplatin significantly inhibited the proliferation of parental cells, while the resistant cell lines HN4/Cis and HN30/Cis exhibited significantly reduced sensitivity to cisplatin, with the resistance factors of more than 5. CYH33 exhibited comparable inhibitory activity in both parental and resistant cells, indicating that CYH33 was able to potently inhibited the proliferation of cisplatin-resistant HNSCC cells (Fig. 1E and Fig S1A). To investigate activity of CYH33 against HNSCC in vivo, the therapeutic efficacy of CYH33 in nude mice bearing HN4 xenografts was evaluated. As shown in Fig. 1F and Fig S1B, administration of CYH33 at 20 mg/kg significantly inhibited the growth of HN4 xenografts, which was accompanied with decreased level of phosphorylated Akt and Ki-67 staining in tumor tissues. No significant loss of body weight was detected in mice during the treatment (Fig. S1B). These results suggested that CYH33 displayed promising therapeutic activity against HNSCC.

### Inhibition of PI3K/AKT pathway by CYH33 was not sufficient for its activity against HNSCC

**Fig. 2:**
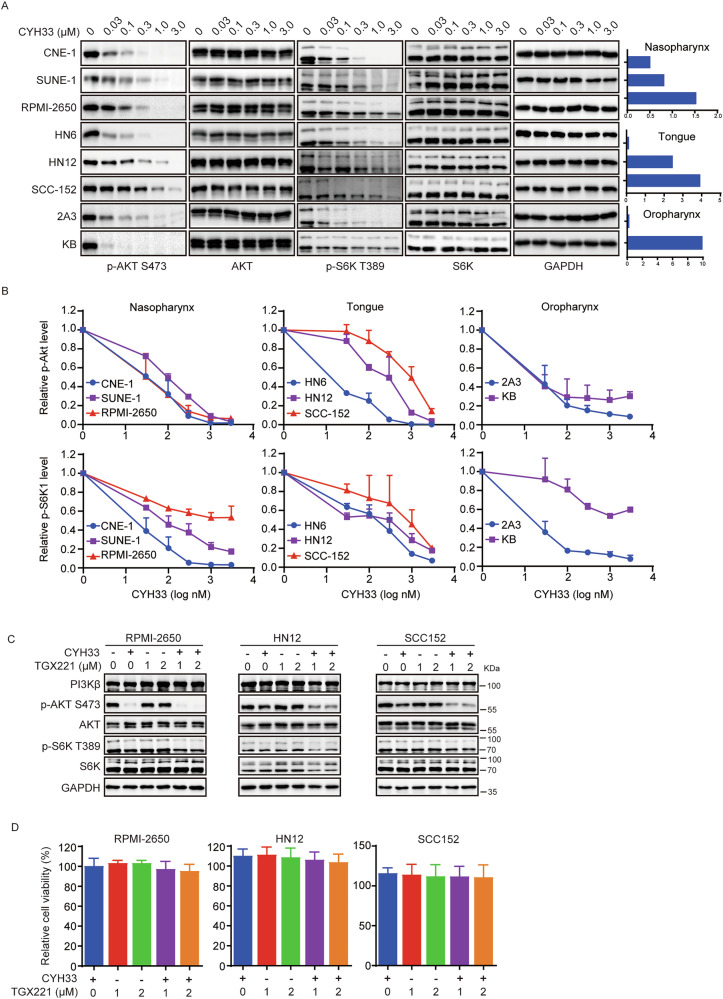
Inhibition of PI3K/AKT pathway by CYH33 was not sufficient for its activity against HNSCC. **A** Representative sensitive and resistant HNSCC cells were treated with indicated concentrations of CYH33 for 1 h. Cell lysates were then subjected to Western blot to detect the indicated proteins. **B** The intensity of the bands in Fig. 2A was quantified by Image lab (*n* = 2). Data were presented as mean + SEM. **C** RPMI-2650, HN12 and SCC-152 cells were exposed to CYH33 (0.1 μM) or TGX221 alone or concurrently for 1 h. Cell lysates were subjected to Western blot with the indicated antibodies. **D** RPMI-2650, HN12 and SCC-152 cells were treated with CYH33 (0.1 μM) or TGX221 alone or concurrently for 72 h. Cell viability was measured by CellTiter-Glo assay (*n* = 2). Data were presented as mean + SEM.

To investigate the mechanism by which CYH33 exhibits its anti-HNSCC activity, we interrogated the effect of CYH33 on the PI3K/Akt signaling in representative sensitive and resistant cells originated from different tissues. As shown in Fig. 2A, B, CYH33 reduced the phosphorylation levels of Akt and S6K dose-dependently in both sensitive and resistant cells. Although inhibition of S6K phosphorylation by the same concentration of CYH33 was more pronounced in sensitive cells originated from nasopharynx, there was no significant difference in the effect on Akt phosphorylation between sensitive (CNE-1 and SUNE-1) and resistant (RPMI-2650) cells. Among tongue squamous carcinoma cells, CYH33 at 0.3 μM potently decreased phosphorylated Akt and S6K in sensitive HN6 cells, while higher concentration was needed to achieve similar effect in resistant HN12 and SCC-152 cells. Though CYH33 completely inhibited Akt phosphorylation at 0.1 μM in KB cells originated from oropharynx, it moderately inhibited the proliferation of KB cells with an IC_50_ > 10 μM. As PI3Kα and PI3Kβ are predominantly expressed in solid tumors, we next detected whether simultaneous inhibiting PI3Kβ would enhance the inhibition on PI3K/Akt pathway as well as cell proliferation. As shown in Fig. 2C, selective inhibition of PI3Kβ by TGX221 farther reduced the phosphorylation levels of Akt and S6K in resistant RPMI-2650/HN12/SCC152 cells. However, TGX221 failed to enhance the activity of CYH33 to inhibit the proliferation of these cells (Fig. 2D). *PIK3CA* mutation has been reported as a potential predictor for the sensitivity of PI3Kα inhibitors, the association between PI3K pathway status and CYH33 activity was also examined. As shown in Figs. S2A, B, the status of PI3K pathway failed to show significant correlation with CYH33 activity in HNSCC cells. These results indicated that inhibition of PI3K/Akt pathway by CYH33 was not sufficient for its activity against HNSCC.

### Proteomics/phosphoproteomics landscape revealed comprehensive regulation of kinome by CYH33

**Fig. 3:**
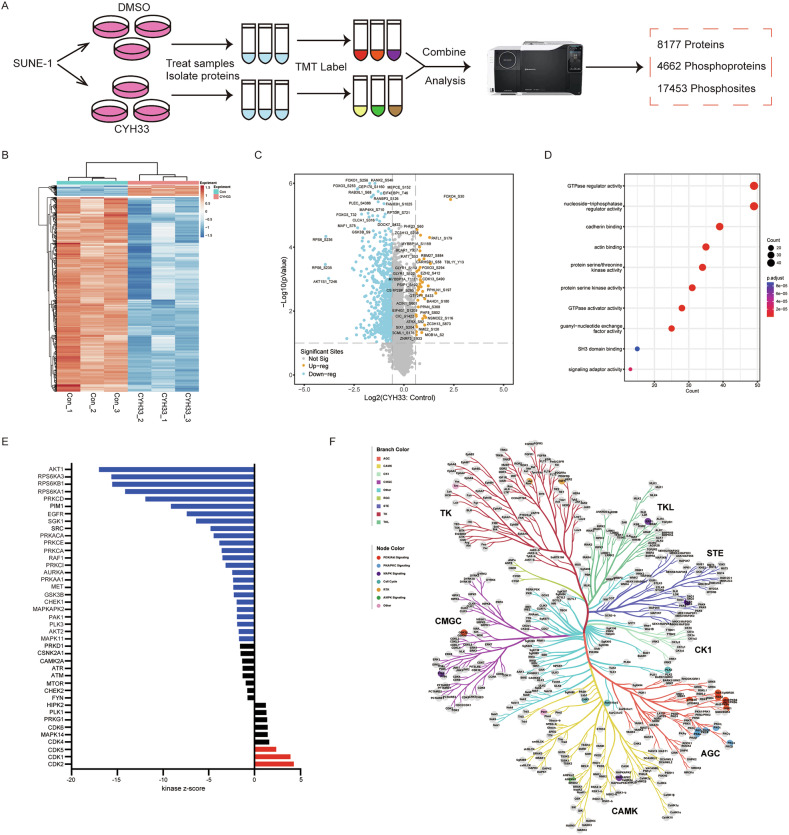
Proteomics/phosphoproteomics landscape revealed comprehensive regulation of kinome by CYH33. **A** The schematic overview of TMT proteomics and phosphoproteomics. Differentially phosphorylated proteins (*p* < 0.05, fold change ≥ 1.5) upon CYH33 treatment were shown in heatmaps (**B**) and Volcano plot (**C**). **D** Gene Ontology enrichment of proteins with significant downregulation in phosphorylation was illustrated in the bubble diagram. **E** Kinase-Substrate Enrichment Analysis of proteins with significant alteration in phosphorylation was performed with Omicsolution database (https://www.omicsolution.com/wkomics). **F** The kinases predicted to be downregulated by CYH33 in (**E**) were shown in Kinome Tree.

PI3K sits at the center of cell signaling, while the effect of inhibiting PI3K on global phosphoproteome remains largely unknown. Tandem-Mass-Tag (TMT) proteomics and phosphoproteomics were employed in sensitive SUNE-1 cells treated with CYH33 for 1 h. A total of 8177 proteins, 4662 phosphorylated proteins, and 17,453 phosphorylation sites were detected in 6 samples (Fig. 3A). The clustering analysis revealed that there was significant difference at protein phosphorylation but not at total protein between control group and CYH33 treatment group (Fig. S3A). We identified 836 protein phosphorylation sites with significant alteration after CYH33 treatment (*p* < 0.05, fold change ≥ 1.5). Among them, phosphorylation of 794 sites was significantly downregulated while that of 42 sites was significantly upregulated (Fig. 3B). Notably, the phosphorylation level of several PI3K downstream effectors, such as Akt, GSK3, RPS6, and FOXO, were significantly downregulated, which was consistent with the cellular targeting PI3K by CYH33 (Fig. 3C). However, the abundance of only 10 proteins significantly changed after CYH33 treatment (*p* < 0.05, fold change ≥ 1.2) (Fig. S3B, C and Table S2), indicating that the alteration in phosphoproteome was not due to the effect on total proteome. The Gene Ontology (GO) enrichment analysis revealed that pathways involved in the activities of GTPases regulator, nucleoside−triphosphatase regulator and serine/threonine protein, etc. have been enriched in SUNE-1 cells upon CYH33 treatment (Fig. 3D). Kinase-Substrate Enrichment Analysis (KSEA) demonstrated that CYH33 significantly downregulated the activity of kinases downstream of PI3K including AKT, S6K, and GSK3β (Fig. 3E). Additionally, the activity of PKA, PKC and several kinases in MAPK pathways was also significantly inhibited by CYH33. We then constructed a kinome tree to visually illustrate the kinase profile affected by CYH33. As shown in Fig. 3F, kinases inhibited by CYH33 were primarily concentrated in PI3K/Akt, PKA/PKC, MAPK, Cell Cycle, RTK, and AMPK pathways, reflecting the complexity of signaling network regulated by CYH33. Collectively, inhibition of PI3K by CYH33 comprehensively altered the phosphoproteomic landscape in HNSCC cells, which may contribute to its anti-proliferative activity.

### CYH33 significantly suppressed the phosphorylation of Erk in sensitive cells

**Fig. 4:**
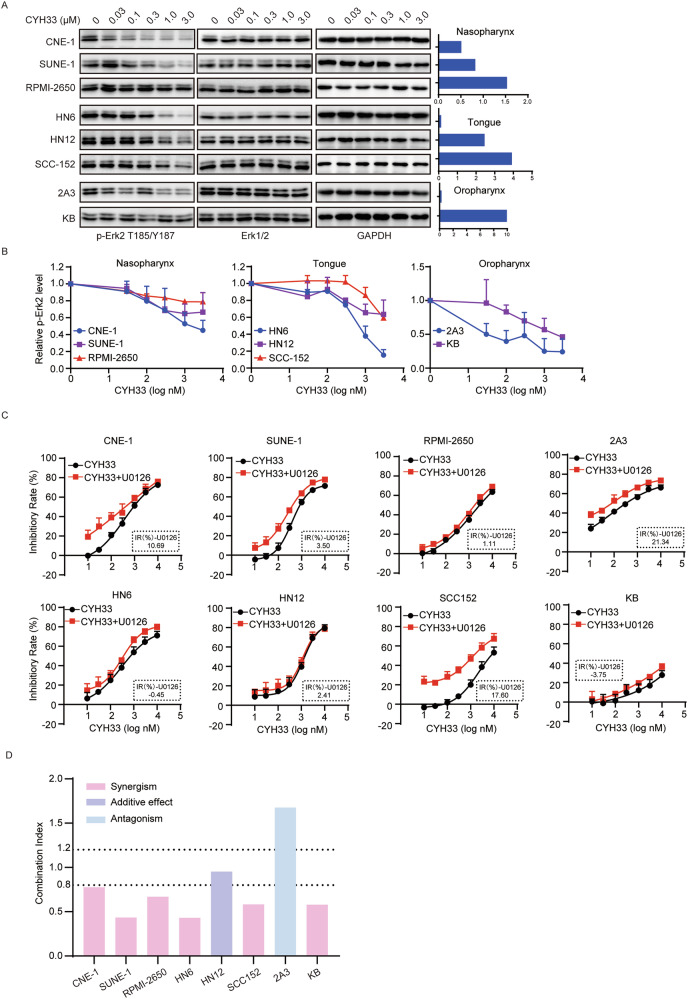
CYH33 significantly suppressed the phosphorylation of Erk in sensitive cells. **A** Representative sensitive and resistant HNSCC cells were treated with indicated concentrations of CYH33 for 1 h. Cell lysates were then subjected to Western blot for the indicated proteins. **B** The intensity of the bands in (**A**) was quantified by Image lab (*n* = 2). Data were presented as mean + SEM. **C**, **D** Representative sensitive and resistant HNSCC cells were treated with serially diluted CYH33 alone or concurrently with 1 μM of U0126 for 72 h. Cell proliferation was measured with SRB assay (**C**) and combination index (CI) values were calculated by the ratio of IC_50_ obtained with CYH33 alone to that obtained with the co-treatment of CYH33 and U0126 (**D**). Data were presented as mean + SD (*n* = 3).

We have previously reported that decrease in phosphorylated Erk indicated the therapeutic efficacy of CYH33 in breast cancer [25]. Similarly, phosphoproteomic analysis showed the phosphorylation of MAPK1/Erk at T185/Y187 was significantly attenuated by CYH33 (Fig. 3C and Table S1). To investigate whether inhibition of Erk phosphorylation is required for the activity of CYH33 in HNSCC cells, the level of phosphorylated Erk in sensitive and resistant cells upon CYH33 treatment was examined. As shown in Fig. 4A, B, CYH33 decreased the phosphorylated Erk in a dose-dependent manner in sensitive CNE-1 and SUNE-1 cells but not in resistant RPMI-2650 cells. In tongue squamous carcinoma cells, 1 μM CYH33 potently decreased phosphorylated Erk in sensitive HN6 cells, while higher dose of CYH33 was required to achieve potent inhibition on the phosphorylation of Erk in resistant HN12 and SCC-152 cells. Similarly, CYH33 at the concentration of 1 μM significantly reduced Erk phosphorylation in sensitive 2A3 cells but not resistant KB cells. These results indicated that the inhibition of Erk phosphorylation was associated with the activity of CYH33 against HNSCC cells derived from the same tissue. MEK inhibitor U0126 enhanced the inhibition of Erk phosphorylation by CYH33 in HNSCC cells (Fig S4). Accordingly, combination of U0126 and CYH33 exhibited a strong synergistic effect in most tested HNSCC cell lines (Fig. 4C, D). Thus, suppressing phosphorylation of Erk contributed to the activity of CYH33 against HNSCC.

### CYH33 attenuated the membrane localization and phosphorylation of GAB1 dependent on PI3K

**Fig. 5:**
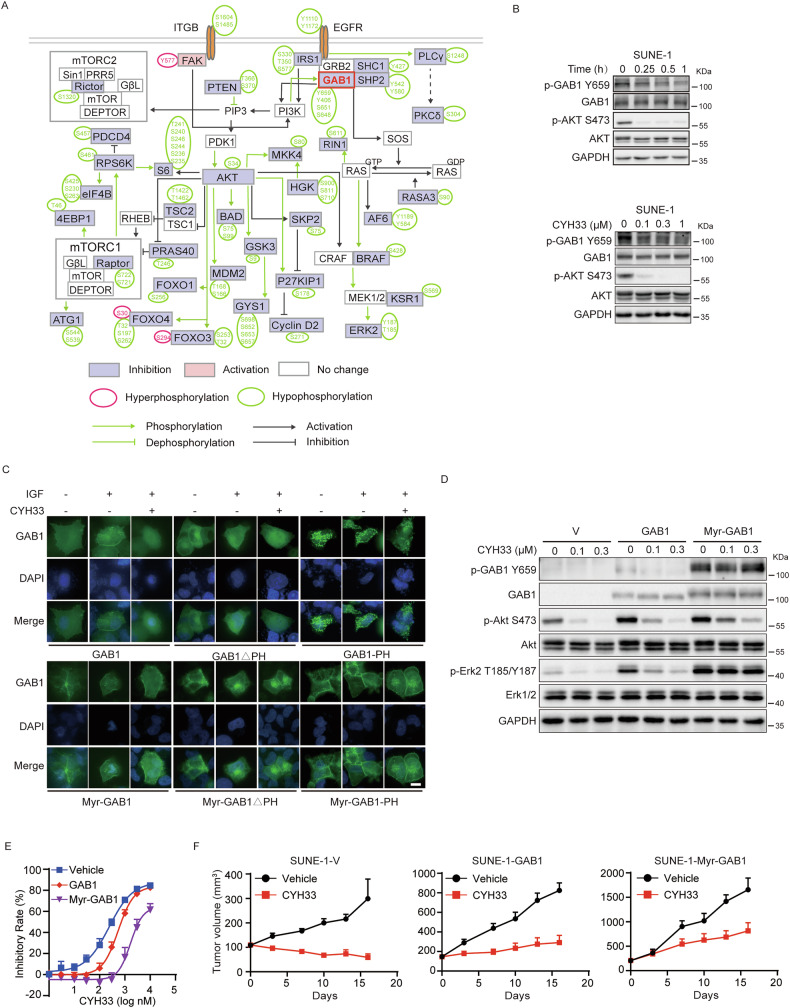
CYH33 attenuated the membrane localization and phosphorylation of GAB1 dependent on PI3K. **A** Signal network analysis of differentially phosphorylated proteins in SUNE-1 cells after CYH33 treatment for 1 h. **B** SUNE-1 cells were treated with 1 μM CYH33 for indicated time or indicated concentrations of CYH33 for 1 h. Cell lysates were then subjected to Western blot with indicated antibodies. **C** SUNE-1 cells transiently transfected with plasmids expressing EGFP-GAB1, EGFP-GAB1ΔPH, or EGFP-GAB1-PH or myristoylated proteins (EGFP-Myr-GAB1, EGFP-Myr-GAB1ΔPH and EGFP-Myr-GAB1-PH) were cultured in serum-free medium for 12 h and pretreated with 1 μM of CYH33 for 1 h before stimulation with IGF (100 ng/ml). Representative images were shown from three independent experiments. Scale bar: 10 μm. SUNE-1 cells transfected with plasmids expressing GAB1, Myr-GAB1 or vehicle control were treated with indicated concentrations of CYH33 for 1 h (**D**) or 72 h (**E**). Cell lysates were subjected to Western blot with indicated antibodies (**D**). Cell proliferation was measured by SRB assay (**E**). **F** Randomly grouped BALB/c nude mice bearing SUNE-1-V, SUNE-1-GAB1, or SUNE-1-Myr-GAB1 xenografts were administrated orally with vehicle control or CYH33 (20 mg/kg) once a day for 16 days (*n* = 6). Tumor volume was measured twice a week. Data were presented as mean + SEM.

To further explore the mechanism of the inhibition of Erk phosphorylation by CYH33, signaling network was constructed with proteins detected with altered phosphorylation to illustrate the reprogramming of signaling transduction mediated by PI3K and MAPK pathways (Fig. 5A). We identified that phosphorylation of the adaptor protein GAB1 at multiple sites (Y659, Y406, S651, S648) were significantly downregulated upon CYH33 treatment. GAB1 plays an important role bridging PI3K and MAPK signaling [26]. Consistently, CYH33 time- and dose-dependently suppressed the phosphorylation level of GAB1 at Y659 in sensitive HNSCC cells, whereas it had little effect in resistant cells (Fig. 5B and Fig. S5A, B). Phosphorylation of GAB1 is mediated by receptor tyrosine kinases (RTKs) upon various stimuli, which is accompanied by the translocation of GAB1 from cytoplasm to cell membrane [27]. It has been reported that the GAB1 membrane localization was associated with the interaction between its PH domain and membrane-bound PIP3 [28, 29]. Indeed, activation of PI3K in SUNE-1 cells with IGF induced translocation of GAB1 to cellular membrane, while the process was blocked by pretreatment of CYH33 (Fig. 5C). Similar results were obtained in cells expressing the fused EGFP-PH domain of GAB1 (GAB1-PH). Meanwhile, translocation of GAB1 failed to be observed in SUNE-1 cells transiently expression of GAB1 deleted with PH domain (GAB1ΔPH) (Fig. 5C). Modification by myristoylation anchors GAB1 to membrane in a PH domain-independent manner [30]. SUNE-1 cells were transfected with plasmids expressing myristoylated GAB1 (Myr-GAB1) or its truncated mutants (Myr-GAB1ΔPH and Myr-GAB1-PH). As shown in Fig. 5C, Myr-GAB1, Myr-GAB1ΔPH and Myr-GAB1-PH constantly localized at the membrane irrespective the stimulation with IGF or CYH33 treatment. These results demonstrated that inhibition of PI3K by CYH33 abrogated cellular localization of GAB1 via its PH domain. As the membrane localization of GAB1 enables the recruitment and activation of downstream signaling including MAPK pathway, we next examined Erk phosphorylation regulated by GAB1 in SUNE-1 cells. As shown in Fig. 5D, enforced expression of GAB1 activated PI3K and MAPK pathway with elevated level of phosphorylated Akt and Erk in SUNE-1 cells, which was inhibited by CYH33 even though the potency was less compared to that in cells transfected with vehicle control. Overexpression of Myr-GAB1 further enhanced the phosphorylation levels of GAB1, Akt and Erk in SUNE-1 cells. Though CYH33 was capable of blocking Akt phosphorylation, the phosphorylation level of GAB1 and Erk remained largely unchanged upon CYH33 treatment in these cells. Accordingly, overexpression of Myr-GAB1 rendered SUNE-1 cells more resistant to CYH33 compared to those transfected with vehicle control or GAB1 (Fig. 5E). Similar results were obtained after alpelisib treatment (Fig. S5C, D). We then evaluated the efficacy of CYH33 against the growth of xenografts derived from SUNE-1-V, SUNE-1-GAB1 or SUNE-1-Myr-GAB1 cells. As shown in Fig. 5F and Fig S5G, forced expression of GAB1 or Myr-GAB1 significantly promoted the tumor growth, with average tumor volumes reaching 300 mm³, 825 mm³, or 1655 mm³ at the end of experiment for SUNE-1-V, SUNE-1-GAB1 or SUNE-1-Myr-GAB1 xenografts respectively. Furthermore, overexpression of Myr-GAB1 or GAB1 rendered SUNE-1 xenografts more resistant to CYH33 compared to those transfected with vehicle control, with T/C values of 48.95%, 35.39% or 19.58% respectively (Fig. 5F and Fig S5E). Accordingly, though CYH33 treatment effectively suppressed Akt phosphorylation, the phosphorylation levels of GAB1 and Erk remained largely unchanged in SUNE-1-GAB1 or SUNE-1-Myr-GAB1 xenografts. The phosphorylation level of Erk even increased in SUNE-1-Myr-GAB1 xenografts after CYH33 administration (Fig. S5F). Taken together, CYH33 not only inhibited the phosphorylation of Akt but also attenuated the membrane localization and phosphorylation of GAB1, resulting in reduced PI3K and MAPK signaling and ultimately inhibiting the proliferation of HNSCC cells in vitro and in vivo.

### Inhibition of GAB1 phosphorylation independent of PI3K potentiated the activity of CYH33 against HNSCC

**Fig. 6:**
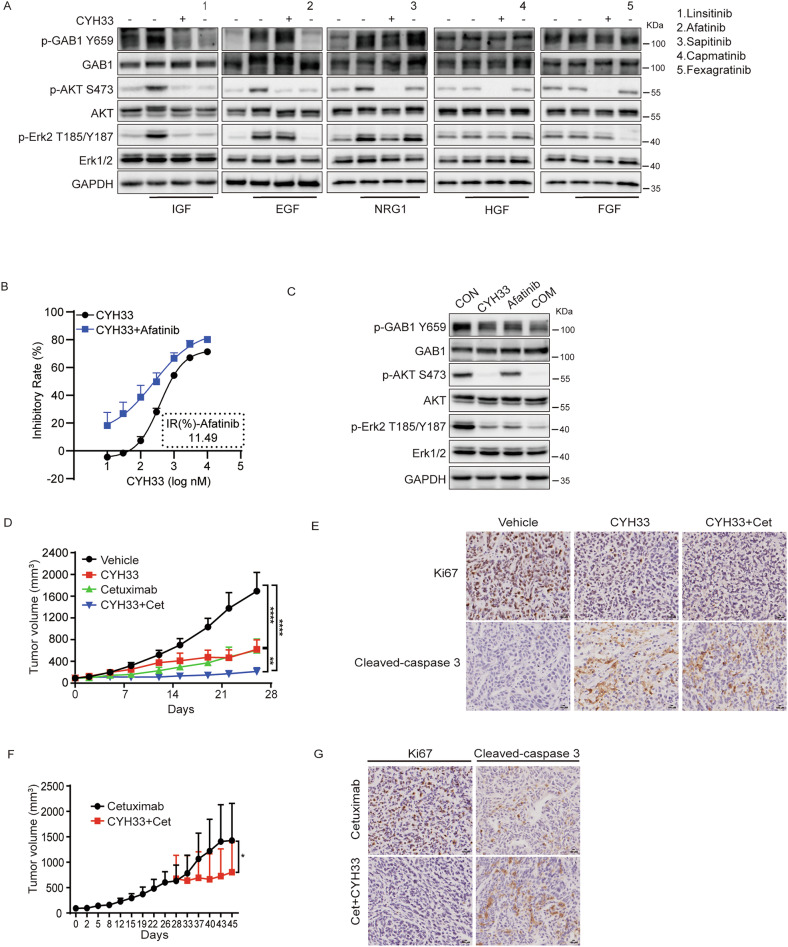
Inhibition of GAB1 phosphorylation independent of PI3K potentiated the activity of CYH33 against HNSCC. **A** SUNE-1 cells cultured in serum-free medium for 12 h were pre-treated with 0.3 μM of CYH33 or 1 μM of respective inhibitor for 1 h before stimulation with indicated growth factors (50 ng/ml). Cell lysates were then subjected to Western blot with indicated protein. **B** SUNE-1 cells were treated with serially diluted CYH33 alone or concurrently with 1 μM Afatinib for 72 h. Cell proliferation was measured with SRB assay. Data were presented as mean + SD (*n* = 3). **C** SUNE-1 cells were treated with 1 μM CYH33 or 1 μM Afatinib alone or concurrently for 1 h. Cell lysates were then subjected to Western blot with indicated antibodies. **D** Randomly grouped BALB/c nude mice bearing SUNE-1 xenografts were administrated with vehicle control, CYH33 (20 mg/kg), Cextuximab (20 mg/kg), or a combination of CYH33 and Cextuximab for 26 days (*n* = 6). CYH33 was administrated orally once a day and Cetuximab was administered intraperitoneally three times a week. Tumor volume was measured twice a week. Data presented are mean + SEM. Differences between the indicated groups were analyzed using two-tailed one-way ANOVA with Tukey multiple group comparison test. **p < 0.01; ****p < 0.0001. **E** Tumor tissues were collected at the end of treatment to detect Ki-67 or cleaved-caspase3. Scale bars, 100 μm. Representative images from each group are shown. **F** Mice treated with Cetuximab for 26 days were divided into two groups to receive Cetuximab alone, or concurrently with CYH33 (20 mg/kg). Tumor volume was measured twice a week. Data presented are mean + SEM. Difference between groups was analyzed using two-tailed unpaired Student’s *t*-test. *p < 0.05. **G** Tumor tissues were collected at the end of treatment to detect Ki-67 or cleaved-caspase 3. Scale bars, 100 μm. Representative images from each group are shown.

Large scale next-generation sequencing has evidenced that several RTKs, such as EGFR, ERBB2, IGF1R, MET and FGFR, are frequently amplified or overexpressed in HNSCC [10]. GAB1 is able to be recruited and activated by multiple RTKs via its proline-rich region, which is not necessarily dependent of PI3K. Thus, inhibition of RTKs that phosphorylate GAB1 independent of PI3K may further potentiate the activity of CYH33 against HNSCC. The RTKs that activated GAB1 independently of PI3K were firstly screened. As shown in Fig. 6A, IGF, EGF and NRG1 effectively activated GAB1 in SUNE-1 cells accompanied by a notable increase in phosphorylation of Akt and Erk, while HGF and FGF exerted little effect on GAB1 activation. CYH33 potently suppressed GAB1 activation induced by IGF and NRG1, suggesting IGFR and ERBB2 activated GAB1 in a PI3K-dependent manner. However, the activation of GAB1 induced by EGF was attenuated by EGFR inhibitor Afatinib but not CYH33, indicating that EGFR induced-GAB1 phosphorylation was independent of PI3K. We next investigated whether concurrent inhibition of EGFR would potentiate the activity of CYH33. As shown in Fig. 6B, Afatinib significantly augmented anti-proliferative efficacy of CYH33 against SUNE-1 cells with a combination index (CI) value of 0.62. Consistently, CYH33 or Afatinib alone decreased phosphorylated GAB1 and Erk in SUNE-1 cells, while concurrent treatment of CYH33 and Afatinib further enhanced these effects (Fig. 6C). We then selected Cetuximab, an anti-EGFR antibody approved for the clinical treatment of HNSCC, combined with CYH33 to evaluate the efficacy against HNSCC in vivo. CYH33 or Cetuximab alone inhibited the growth of SUNE-1 xenografts with a T/C of 35.8% or 35.4% respectively, while concurrent administration of CYH33 and Cetuximab synergistically attenuated tumor growth, yielding a T/C of 12.3% (Fig. 6D and Fig S6A). The enhanced efficacy of the combination was also reflected by decreased staining of Ki-67 and elevated staining of cleaved-caspase 3 in tumor tissues collected at the end of treatment (Fig. 6E). Accelerated tumor growth was observed in mice treated with Cetuximab monotherapy for 26 days, indicating SUNE-1 tumor may be acquiring resistance to the treatment. To investigate whether CYH33 would overcome the resistance, mice with Cetuximab monotherapy were divided into two groups to receive Cetuximab alone or concurrently with CYH33. As shown in Fig. 6F and Fig S6B, tumor continued to grow during treatment of Cetuximab alone, while the combination of CYH33 and Cetuximab significantly inhibited the tumor growth. Accordingly, the combined treatment decreased staining of Ki-67 and elevated staining of cleaved-caspase 3 in tumor tissues compared to Cetuximab treatment alone (Fig. 6G). Thus, concurrent inhibition of EGFR synergistically potentiated the activity of CYH33 against HNSCC and CYH33 may overcome the adaptive resistance to the therapy targeting EGFR.

## Discussion

**Fig. 7:**
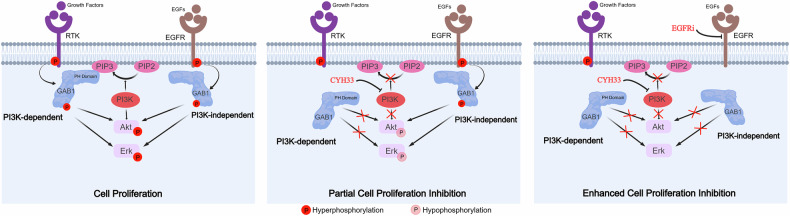
PI3K-dependent GAB1/Erk phosphorylation rendered HNSCC cells sensitive to CYH33. A proposed scheme representing the mechanism of CYH33 executed its anti-HNSCC effect. CYH33 inhibited the proliferation of HNSCC cells via attenuating PI3K-dependent GAB1 membrane localization and phosphorylation, which resulted in reduced activity of PI3K and MAPK pathway. Further inhibition of GAB1 phosphorylation independent of PI3K potentiated the activity of CYH33 against HNSCC.

The hyperactivation of the PI3K pathway has been well-characterized as a pathological hallmark in HNSCC, making PI3K inhibition as an emerging therapeutic strategy for this deadly disease. In this study, we found that a novel PI3Kα-selective inhibitor CYH33 displayed promising therapeutic activity against HNSCC. Inhibition of PI3K/Akt pathway by CYH33 was not sufficient for its activity against HNSCC. Leveraging TMT-phosphoproteomics technology, we revealed that CYH33 attenuated the membrane localization and phosphorylation of GAB1 dependent on its PH domain, which resulted in reduced phosphorylation of Erk and ultimately the proliferation of HNSCC cells. Meanwhile, activation of EGFR induced GAB1 phosphorylation independent of PI3K. Concurrent inhibition of EGFR synergistically potentiated the activity of CYH33 against HNSCC (Fig. 7).

The limited therapeutic options highlight the urgent need for the development of novel targeted therapies for HNSCC. We found that the highly selective PI3Kα inhibitor CYH33 exhibited potent activity against HNSCC in vitro and in vivo, which is being evaluated in advanced HNSCC patients. Currently, several pan-PI3K inhibitors and isoform-selective PI3K inhibitors have advanced to clinical evaluation in HNSCC and preliminary efficacy has been observed. The pan-PI3K inhibitor Buparlisib combined with paclitaxel demonstrated improved progression-free survival in the treatment of recurrent or metastatic HNSCC patients [14]. However, the adverse effects linked to pan-class PI3K inhibition have been observed in a subset of patients, indicating isoform selectivity may improve the therapeutic window. As *PIK3CA* is the most frequently altered gene in PI3K pathway in HNSCC, specific targeting PI3Kα might achieve enhanced therapeutic efficacy with lower toxicity. Alpelisib, the first approved PI3Kα-selective inhibitor, in combination with Cetuximab, has demonstrated safety and efficacy as a radiosensitizing regimen for locally advanced HNSCC patients [31]. In comparison to alpelisib, CYH33 displayed superior anti-HNSCC activity in all tested HNSCC cell lines, underscoring its therapeutic potential for this malignancy. Acquired resistance remains a critical challenge in the treatment of HNSCC. Most HNSCC patients relapsed after chemotherapy or Cetuximab treatment [32, 33]. We also found that CYH33 demonstrated potent anti-proliferative efficacy against cisplatin-resistant HNSCC cells and could overcome acquired resistance to Cetuximab in SUNE-1 xenografts, which further supported the potential of PI3Kα inhibitors for HNSCC treatment. Nevertheless, the therapeutic efficacy of PI3Kα inhibitors in HNSCC remains to be validated in clinical studies.

Phosphoproteomics serves as a powerful approach for characterizing alterations in cellular phosphorylation signaling, providing valuable insights into the antitumor mechanisms of kinase inhibitors [34, 35]. However, studies on the regulation of phosphorylation signaling network by PI3K inhibitors remain limited. In this study, we for the first time employed TMT-phosphoproteomics to uncover the alterations in phosphoproteomic landscape of HNSCC cells upon PI3K inhibition. We found that CYH33 significantly suppressed the phosphorylation level of PI3K downstream effectors including Akt, GSK3, RPS6 and FOXO, confirming target engagement of PI3Kα inhibitors. We also observed that CYH33 significantly suppressed phosphorylation of Erk in sensitive cells and further inhibition of Erk phosphorylation enhanced the activity of CYH33 against HNSCC. In consistency with this finding, our previous work has demonstrated that simultaneous inhibition of the phosphorylation of Akt and Erk is essential for CYH33 to exert its activity against breast cancer [25]. Moreover, activating mutation of *HRAS* conferred resistance to CYH33 in esophageal squamous carcinoma cells by activating Erk [36]. Collectively, these findings indicated that inhibition of MAPK pathway might be indispensable for the antitumor efficacy of PI3K inhibitors. PI3K inhibitors have been reported to exert antitumor activity through the induction of cell cycle arrest [37, 38]. Consistently, CYH33 treatment significantly reduced the activity of multiple cell cycle-associated kinases, suggesting CYH33 might hinder cell cycle progression in HNSCC cells. Notably, CYH33 treatment also attenuated the activity of PKA and PKC in sensitive cells. Given the established roles of PKA and PKC in promoting HNSCC tumorigenesis [39–41], our study provided important clues indicating the potential crosstalk between PI3K and PKC/PKA signaling. Further investigation is warranted to elucidate the role of reduced PKC/PKA activity in the action of PI3K inhibitors against HNSCC.

The phosphorylation of Erk has been demonstrated to correlate with the activity of CYH33 against breast cancer [25], ESCC [36] and HNSCC, but the precise mechanisms underlying how PI3Kα inhibitors suppressed Erk phosphorylation remained unclear. In this study, we identified that GAB1 may function as a key mediator responsible for the concurrent inhibition of Akt and Erk phosphorylation by CYH33. It has been reported that the recruitment of GAB1 to the cellular membrane via its PH domain interacting with membrane-bound PIP3 is critical for its phosphorylation by RTKs [28, 29, 42]. Similarly, our findings showed that inhibition of PI3Kα reduced the membrane localization and phosphorylation of GAB1, which depended on its PH domain. Furthermore, inhibition of Erk phosphorylation by PI3Kα inhibitor was revealed to be mediated by GAB1, as evidenced by the observation that the phosphorylation of GAB1 and Erk remained largely unchanged upon CYH33 treatment in HNSCC cells and xenografts overexpressing myristoylated GAB1. However, GAB1 can also be recruited to membrane and phosphorylated by RTKs through a PI3K-independent manner. Accordingly, we found that EGFR activation induced GAB1 phosphorylation via a PI3K-independent pathway, potentially contributing to the intrinsic resistance of HNSCC cells to CYH33. Thus, concurrent inhibition of EGFR synergistically potentiated the activity of CYH33 against HNSCC accompanied with enhanced inhibition on phosphorylation of GAB1 and Erk. However, the mechanism by which EGF/EGFR signaling triggered GAB1 membrane localization and phosphorylation independent of PI3K has not been fully elucidated. The recruitment of growth factor receptor bound protein 2 (GRB2) to RTKs was reported to be sufficient to induce GAB1 phosphorylation [43, 44]. Further investigation is warranted to determine whether GRB2 mediated the PI3K-independent GAB1 phosphorylation in HNSCC cells. Nevertheless, our findings revealed PI3K-dependent GAB1/Erk phosphorylation rendered HNSCC cells sensitive to CYH33 and also proposed a rational combination regimen to further potentiate the efficacy of CYH33.

In summary, we found that PI3Kα-selective inhibitor CYH33 displayed potent activity against HNSCC, which was associated with inhibition of Akt signaling as well as attenuation of PI3K-dependent GAB1/Erk phosphorylation. Co-targeting EGFR that induced GAB1/Erk phosphorylation independent of PI3K potentiated the activity of CYH33 against HNSCC. These findings uncovered the mechanistic rationale of CYH33 against HNSCC and its combination with EGFR inhibitors for the therapy of HNSCC.

## Supplementary information

Supplementary files

Supplementary Table S1

Supplementary Table S2

WB raw data

## Data Availability

All research data supporting the findings of this study are available upon reasonable request by readers.
