# Supplementary material for: PI3K-dependent GAB1/Erk phosphorylation renders head and neck squamous cell carcinoma sensitive to PI3Kα inhibitors
Source: Cell Death Dis. 2025 Jun 18;16(1):457. doi: 10.1038/s41419-025-07767-x (PMC12177050; doi:10.1038/s41419-025-07767-x)
Supplement: Supplementary file 1 — Supplementary files [file 41419_2025_7767_MOESM1_ESM.docx]

**Supplementary Materials**

**Supplementary Figures**


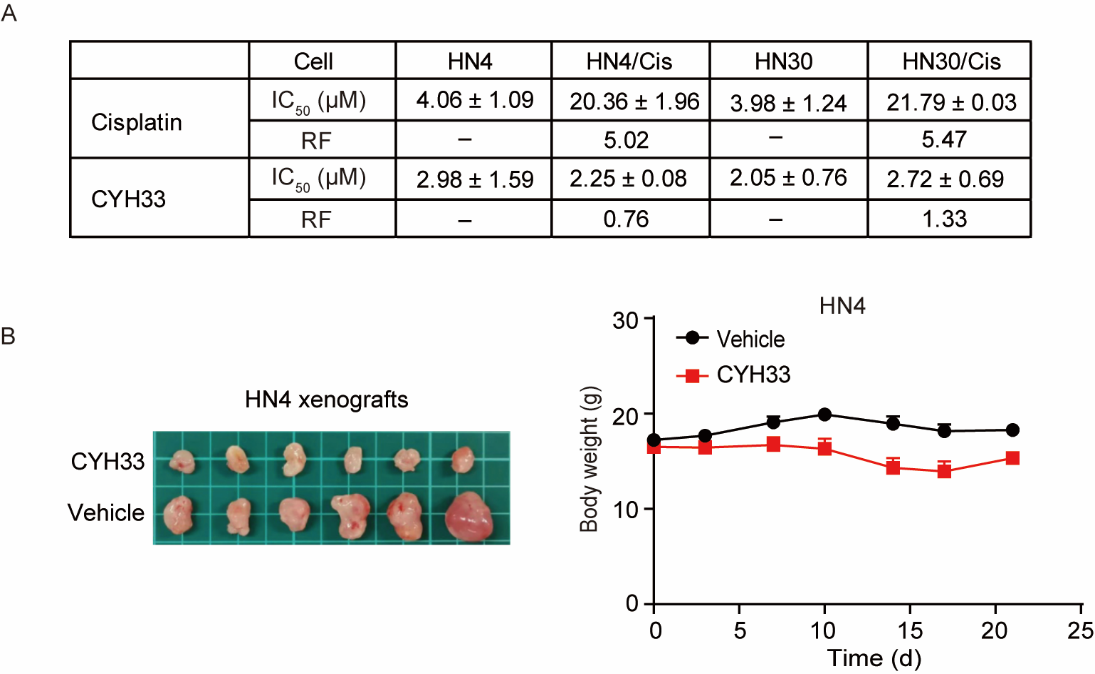


**Figure S1 CYH33 displayed promising therapeutic activity against HNSCC**

**A** Resistance factor (RF) was calculated by the ratio of IC_50_ obtained in Cisplatin-resistant cells and that in parental cells (n = 3). **B** Randomly grouped BALB/c nude mice bearing HN4 xenografts were administrated orally with vehicle control or CYH33 (20 mg/kg) once a day for 21 days (n = 6). Images of xenografts collected from BALB/c nude mice at the end of treatment. Body weight was measured twice a week. Data were presented as mean + SEM.


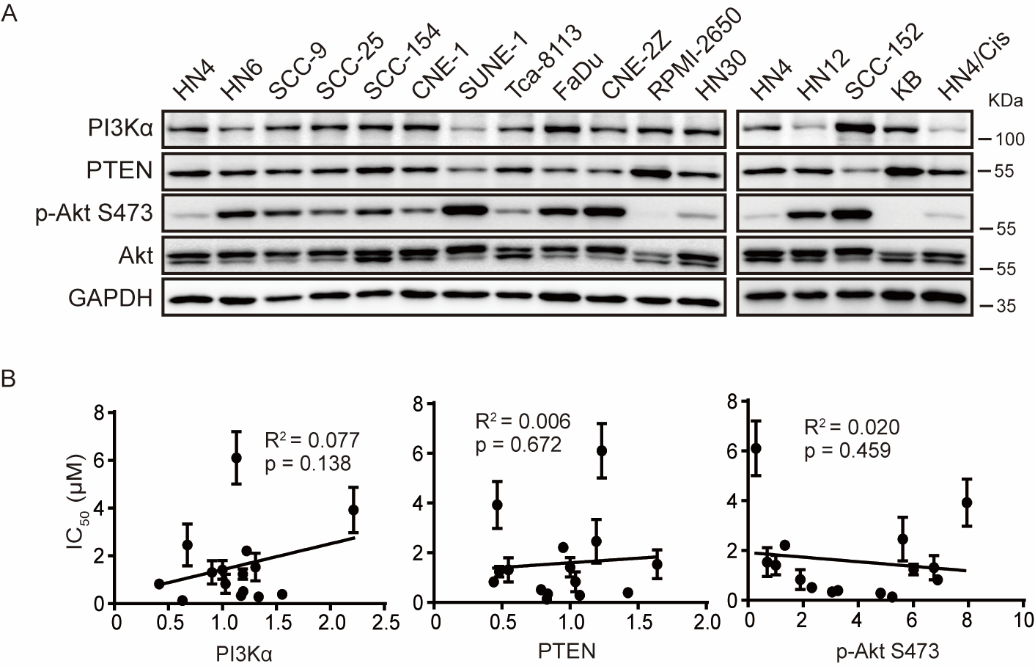


**Figure S2 Inhibition of PI3K/AKT pathway by CYH33 was not sufficient for its activity against HNSCC**

**A** Cell lysates of HNSCC cells were harvested and subjected to Western blot with the indicated antibodies. **B** Pearson correlation analysis of the IC_50_ values of CYH33 and relative protein level of PI3Kα, PTEN or p-Akt S473 was performed among the HNSCC cell lines.


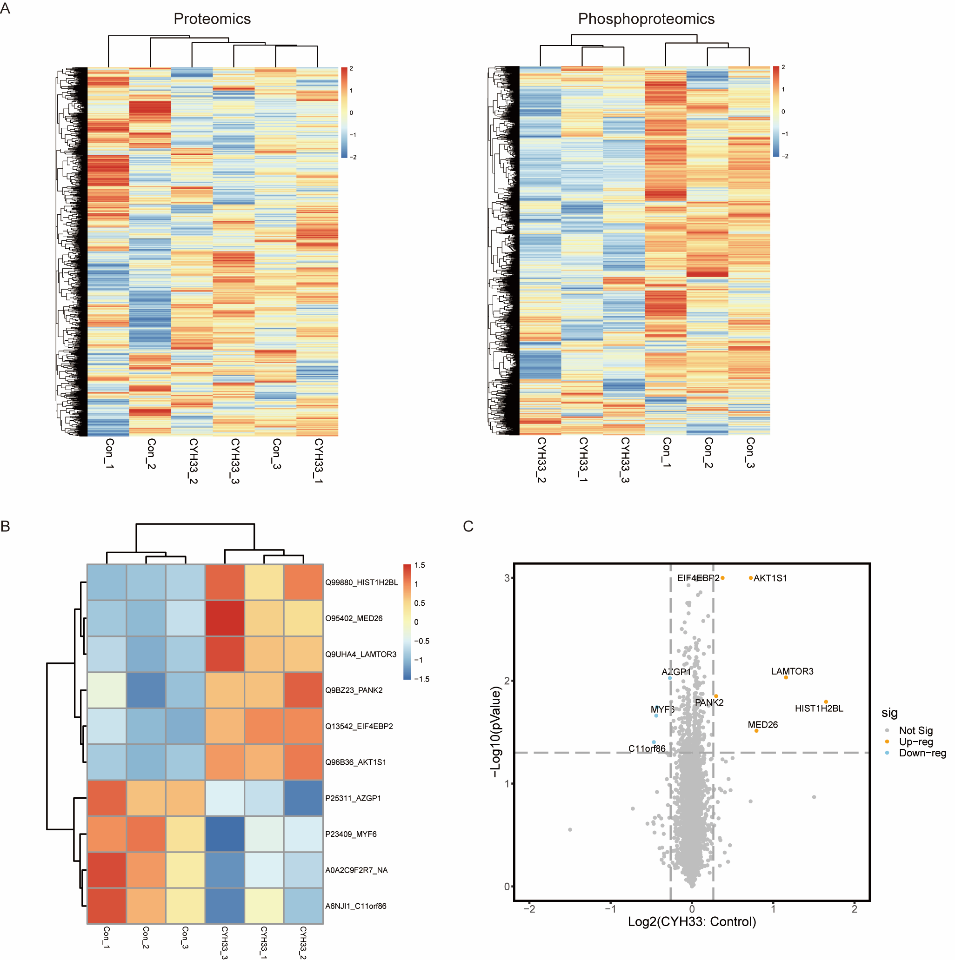


**Figure S3 Proteomics/phosphoproteomics landscape revealed comprehensive regulation of** **kinome by CYH33**

**A** The clustering analysis of 17,453 phosphorylation sites and 8,177 proteins between control group and CYH33-treated group. **B-C** Differentially expressed proteins (p < 0.05, fold change ≥ 1.2) upon CYH33 treatment were shown in heatmaps (B) and Volcano plot (C).


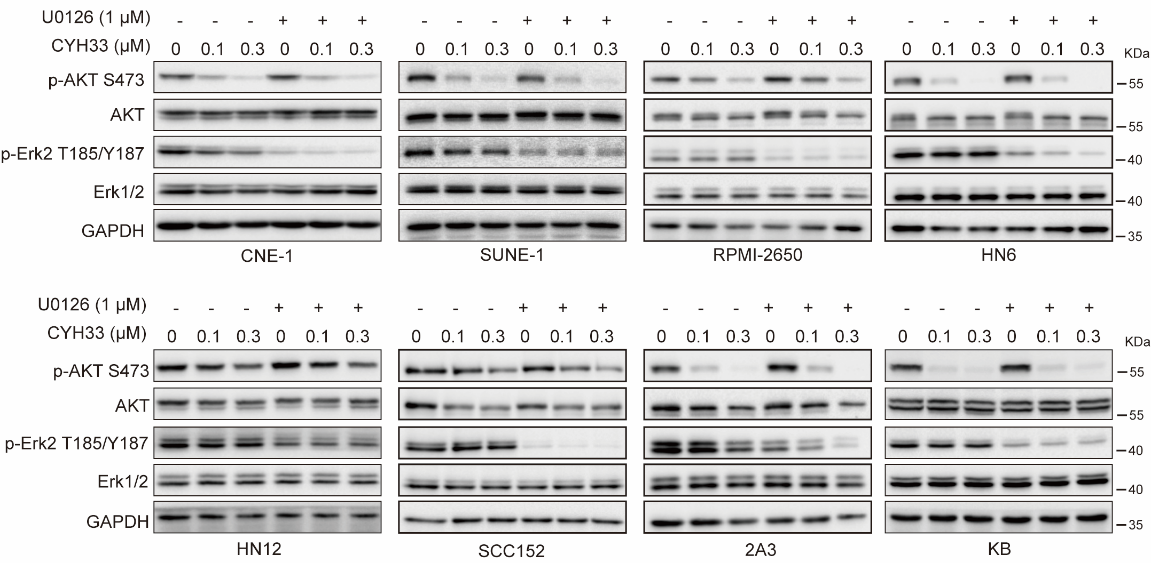


**Figure S4 CYH33 significantly suppressed the phosphorylation of Erk in sensitive cells**

Representative sensitive and resistant HNSCC cells were exposed to CYH33 or U0126 (1 μM) alone or concurrently for 1 h. Cell lysates were then subjected to Western blot with the indicated antibodies.


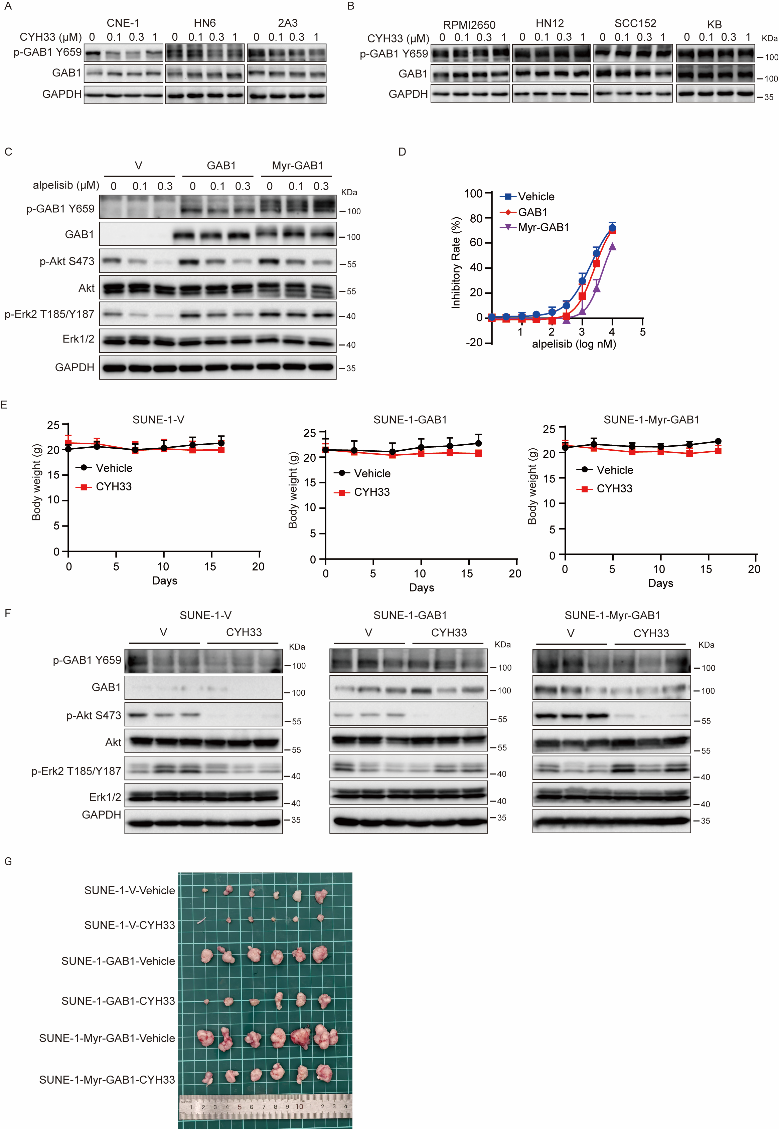


**Figure S5 CYH33 attenuated the membrane localization and phosphorylation of GAB1 dependent on PI3K**

**A-B** Representative sensitive (A) and resistant (B) HNSCC cells were exposed to CYH33 for 1 h. Cell lysates were then subjected to Western blot with the indicated antibodies. **C-D** SUNE-1 cells transfected with plasmids expressing GAB1, Myr-GAB1 or vehicle control were treated with indicated concentrations of alpelisib for 1 h (C) or 72 h (D**)**. Cell lysates were subjected to Western blot with indicated antibodies (C). Cell proliferation was measured by SRB assay (D). **E** Randomly grouped BALB/c nude mice bearing SUNE-1-V, SUNE-1-GAB1, or SUNE-1-Myr-GAB1 xenografts were administrated orally with vehicle control or CYH33 (20 mg/kg) once a day for 16 days (n = 6). Body weight was measured twice a week. Data were presented as mean + SEM. **F** Mice bearing indicated tumor received a single dose of CYH33 (20 mg/kg) and were sacrificed 2 h post administration. Tumor tissues were collected for Western blotting to detect indicated proteins. **G** Images of xenografts collected at the end of treatment.


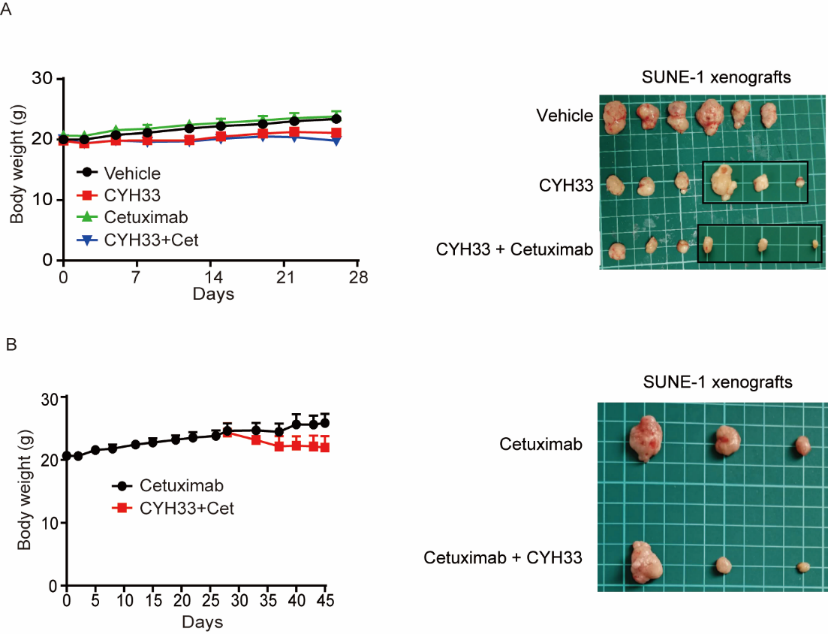


**Figure S6 Inhibition of GAB1 phosphorylation independent of PI3K potentiated the activity of CYH33 against HNSCC**

**A** Randomly grouped BALB/c nude mice bearing SUNE-1 xenografts were administrated with vehicle control, CYH33 (20 mg/kg), Cextuximab (20 mg/kg), or a combination of CYH33 and Cextuximab for 26 days (n = 6). CYH33 was administrated orally once a day and Cetuximab was administered intraperitoneally three times a week. Body weight was measured twice a week. Data presented are mean + SEM. Images of xenografts collected at the end of treatment. The xenografts in the black box were collected after a 24-hour (CYH33) or 48-hour (CYH33 + Cextuximab) interval due to the pharmacokinetics and pharmacodynamics study. **B** Mice treated with Cetuximab for 26 days were divided into two groups to receive Cetuximab alone, or concurrently with CYH33 (20 mg/kg). Body weight was measured twice a week. Data presented are mean + SEM. Images of xenografts collected at the end of treatment.

**Supplementary Tables**

**Table S1. Differentially protein phosphorylation sites (p < 0.05, fold change ≥ 1.5) upon CYH33 treatment.**

**Table S2. Differentially expressed proteins (p < 0.05, fold change ≥ 1.2) upon CYH33 treatment.**
