# Supplementary material for: PI3K-dependent GAB1/Erk phosphorylation renders head and neck squamous cell carcinoma sensitive to PI3Kα inhibitors
Source: Cell Death Dis. 2025 Jun 18;16(1):457. doi: 10.1038/s41419-025-07767-x (PMC12177050; doi:10.1038/s41419-025-07767-x)

**Figure 1F**

**
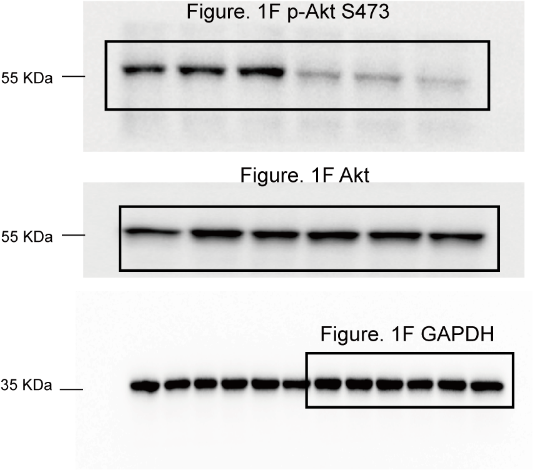
**

**Figure 2A**

**
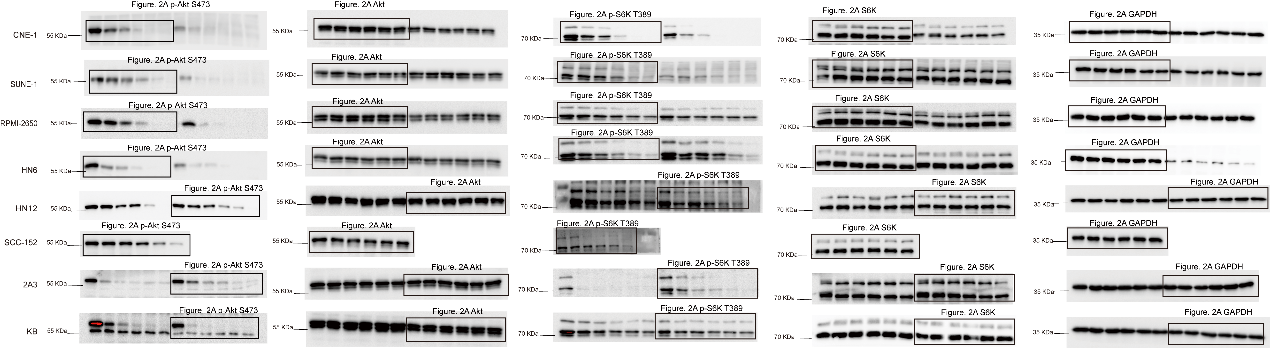
**

**Figure 2C**

**
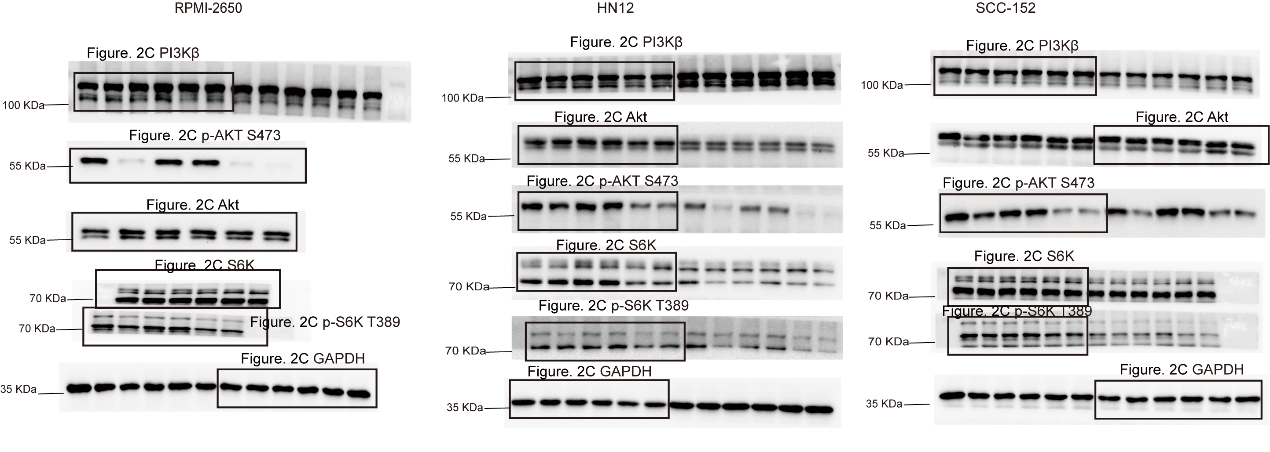
**

**Figure 4A**

**
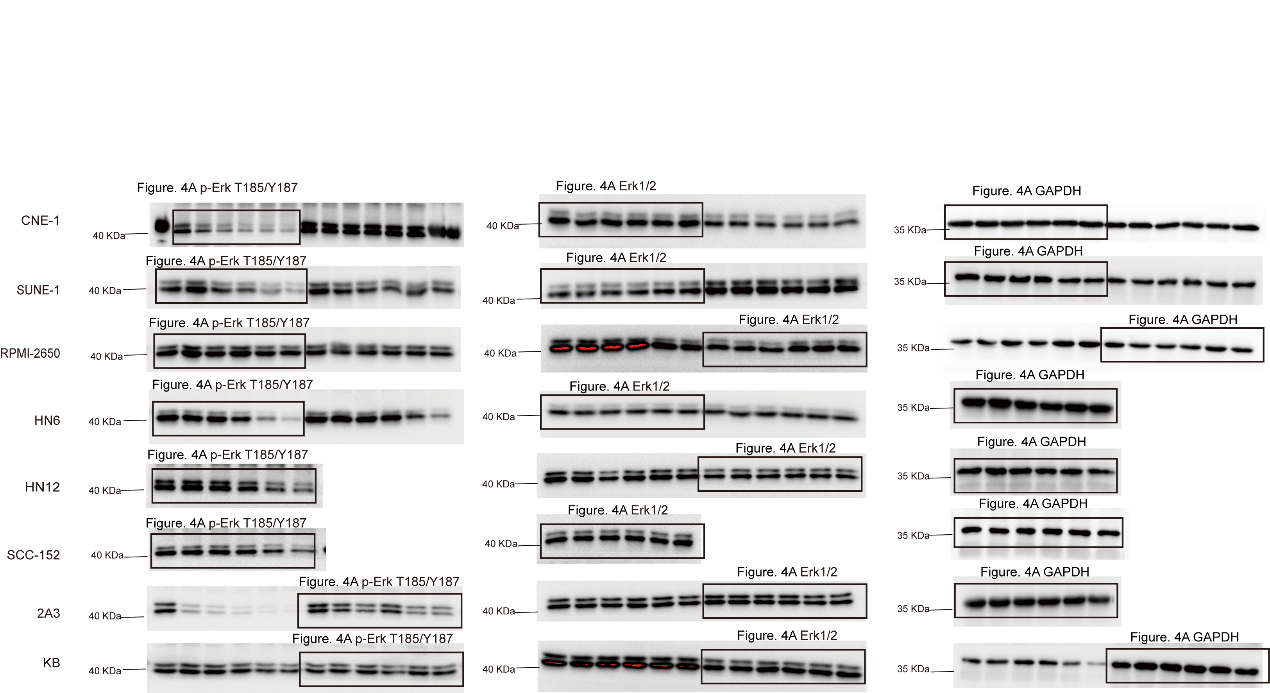
**

**Figure 5B**


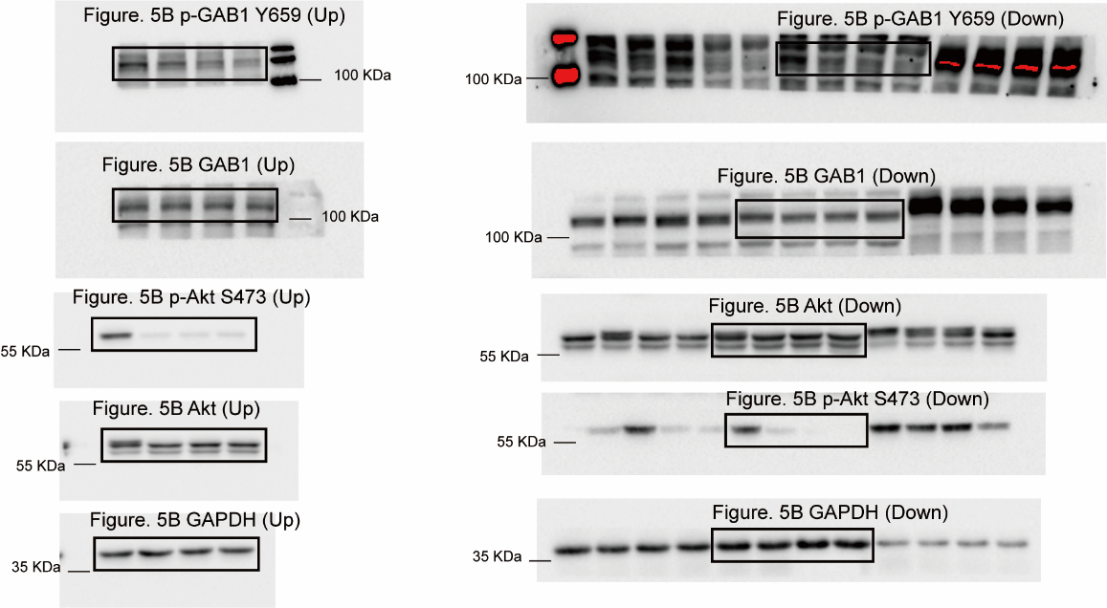


**Figure 5D**


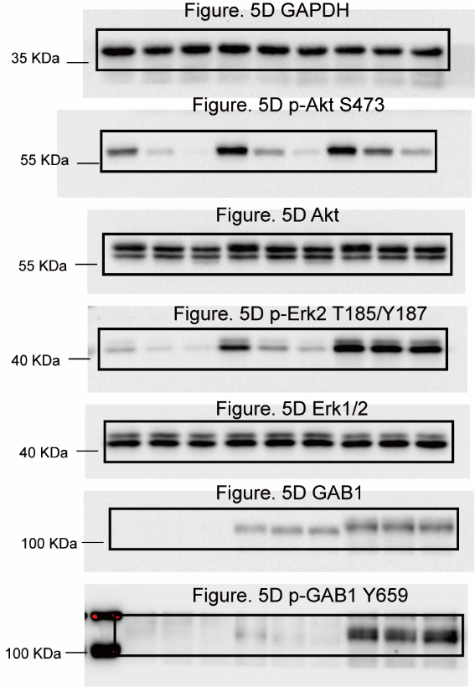


**Figure 6A**


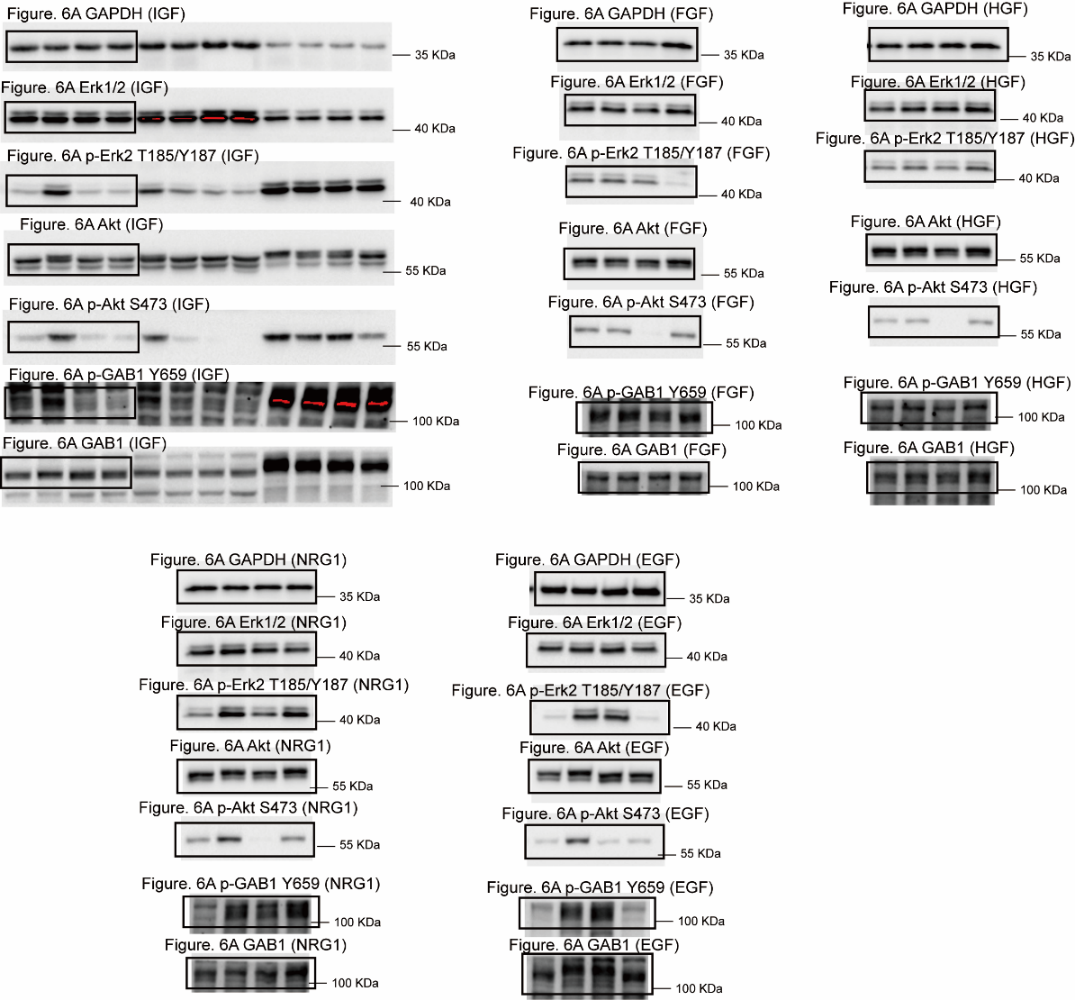


**Figure 6C**


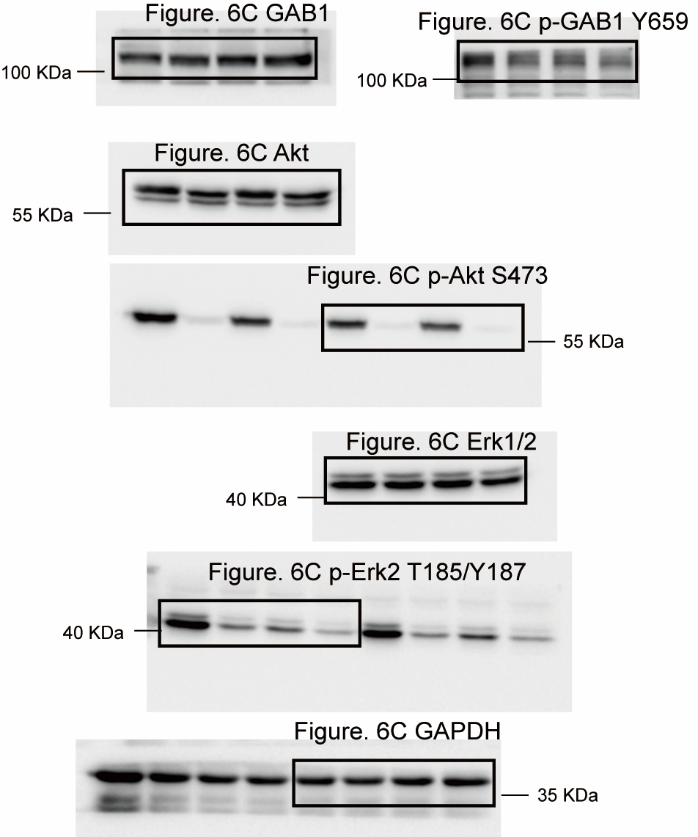


**Figure S2A**

**
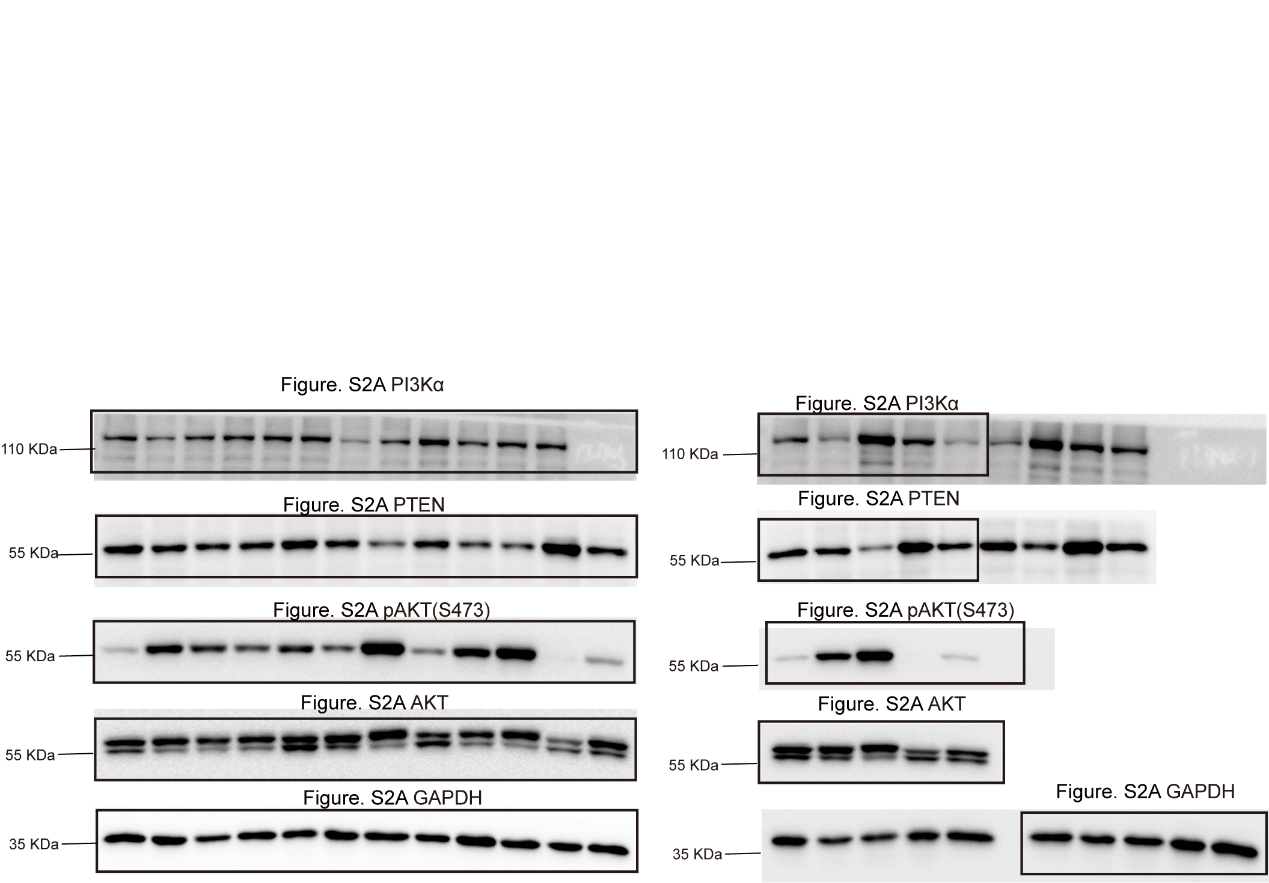
**

**Figure S4**


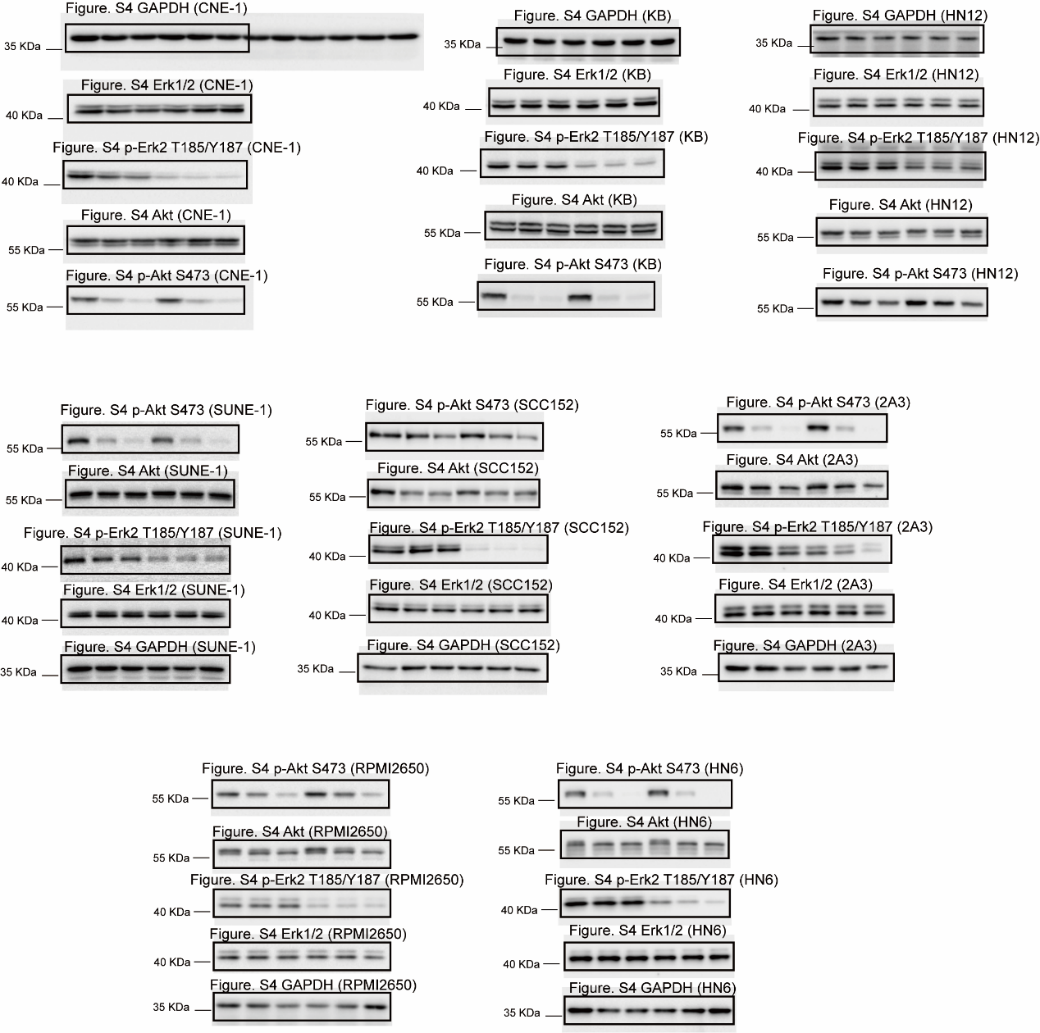


**Figure S5A**


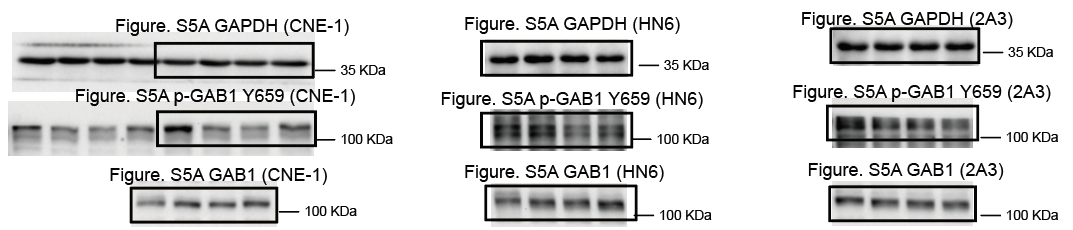


**Figure S5B**


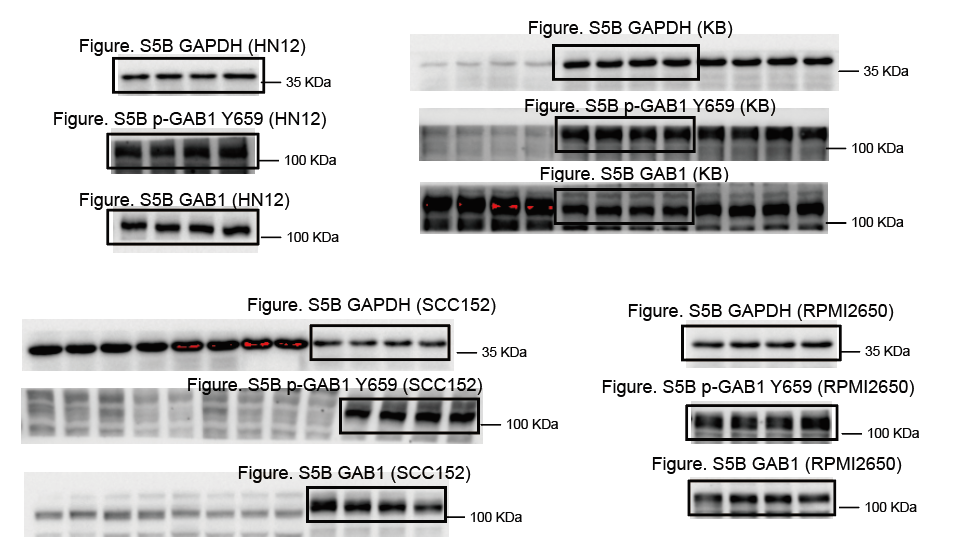


**Figure S5C**


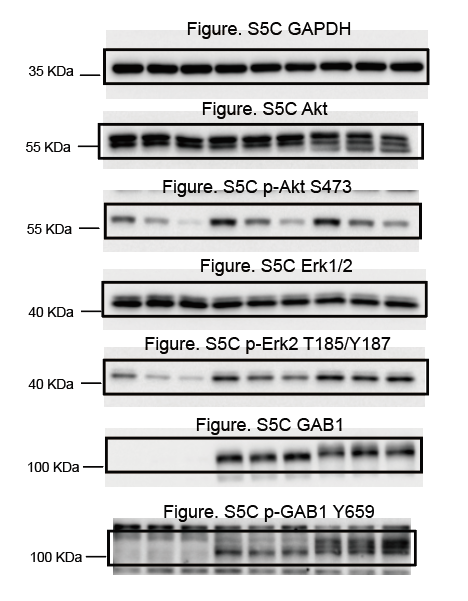


**Figure S5F**


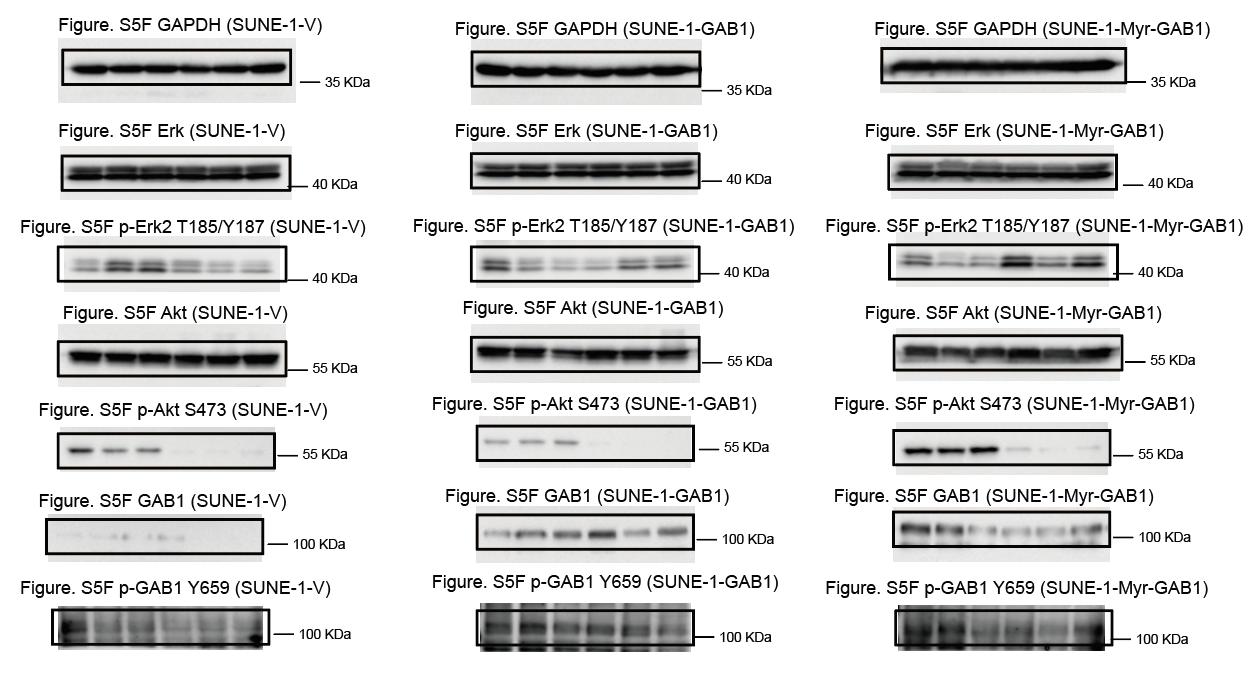

Supplement: Supplementary file 4 — WB raw data [file 41419_2025_7767_MOESM4_ESM.docx]
